# Efficacy and Safety of Carbetocin Versus Misoprostol in Cesarean Section: A Systematic Review and Meta-Analysis

**DOI:** 10.7759/cureus.32901

**Published:** 2022-12-24

**Authors:** Ebraheem Albazee, Moudhi Sadan, Abdulaziz M Alenezi, Abdulrahman N Almutairi, Maryam M Alenezi, Lolwa E Almonayea

**Affiliations:** 1 Department of Internship, Kuwait Institute for Medical Specializations, Kuwait City, KWT; 2 General Medicine, Alsabah Hospital, Kuwait, KWT

**Keywords:** meta-analysis, cesarean section (cs), postoperative blood loss, misoprostol, carbetocin

## Abstract

In the absence of comprehensive data investigating carbetocin versus misoprostol for reducing postpartum hemorrhage (PPH) during cesarean section (CS), we performed this investigation to compare the efficiency and side events of carbetocin versus misoprostol in the protection and reduction of PPH for women who underwent CS. From inception to September 2022, we depended on searching through various databases for eligible trials involving Cochrane, Web of Science, PubMed, Scopus, and Google Scholar. From the efficacy prospect, we found that carbetocin substantially decreased intraoperative blood loss (p<0.001), hemoglobin/hematocrit levels (p<0.001), and the need for blood transfusion (p=0.002)/additional surgical interventions (p=0.003) than misoprostol. However, we revealed no substantial variation between both drugs for the need for additional uterotonic agents (p=0.08). From the safety prospect, we found that incidences of fever (p=0.002), heat sensation (p=0.007), metallic taste (p=0.01), and shivering (p=0.0002) were lower in carbetocin administration than in misoprostol. However, headache (p=0.34) and palpitation (p=0.11) incidences revealed no substantial variation between both drugs. In conclusion, from the efficacy and safety prospect, for women who underwent CS, carbetocin is more effective and safer in preventing and reducing PPH than misoprostol.

## Introduction and background

Cesarean section (CS) is among the most often performed major procedures on women globally [1], and its prevalence is rising, particularly in high- and middle-income nations. Although the World Health Organization (WHO) advised a CS rate of 10% to 15% to reduce both maternal and newborn death ratios [2], the prevalence has increased significantly, particularly in Egypt, reaching 52% of all deliveries [3].

CS-related postpartum hemorrhage (PPH) is a leading factor in maternal death [4], especially when the female loses more blood than 500 ml the day after a natural birth or more than 1000 ml after a CS [5]. Uterine atony is the underlying cause of PPH in almost 70% of instances [6,7]. The WHO encourages the active management of the third stage of labor and the introduction of uterotonic drugs as PPH prophylaxis in all women [8]. However, studies have revealed that 6%-16% of women still suffer hemorrhage over 500 ml despite the use of preventative medicines [9]. As a result, it is crucial to administer uterotonic medications during CS to reduce the incidence of PPH.

Numerous uterotonic medications, such as oxytocin, misoprostol, and carbetocin, were recommended to reduce bleeding during CS. Misoprostol, an analog of prostaglandin, has a strong uterotonic impact. It is affordable, viable at regular temperatures, and causes minimal adverse reactions [10]. When taken orally, vaginally, sublingually, recto-rectally, or buccally, it is well absorbed [11]. Since it enhances the frequency and strength of uterus contractility during labor, it is helpful in both the prevention and treatment of PPH [12].

Carbetocin is an oxytocin analog that has undergone structural changes to lengthen its half-life and extend its therapeutic action [13]. It is recommended to prevent uterine atony and PPH after CS. Carbetocin is injected intravenously to cause cyclic uterine contractility, which continues for around one hour; however, an intramuscular injection greatly extends this activity to 120 minutes [14]. It has been connected to a substantial reduction in the need for other uterotonic drugs and uterus massaging after vaginal childbirth [15].

In the absence of comprehensive data investigating carbetocin versus misoprostol for reducing PPH during CS, w we performed this investigation to compare the efficiency and side events of carbetocin versus misoprostol in the protection and reduction of PPH for women who underwent CS.

## Review

Methods

Sources of Data and Study Selection

The study protocol was not retrospectively recorded in the International Prospective Register of Systematic Reviews (PROSPERO).This study adhered to the guidelines in the Preferred Reporting Items for Systematic Reviews and Meta-Analyses (PRISMA) statement [16] and the Cochrane Handbook for Systematic Reviews of Interventions [17]. Due to the study design, we did not need formal approval from ethics for our work.

From the inception to September 2022, we depended on searching through various databases for eligible trials, involving Cochrane, Web of Science, PubMed, Scopus, and Google Scholar. We adopted the following search strategy and its related terms: (“cesarean section” OR CS OR “C-section” OR “abdominal delivery”) AND (misoprostol OR “novo misoprostol” OR “apo misoprostol” OR cytotec OR “SC30249” OR “SC29333” OR glefos OR misodel OR mysodelle OR misotac) AND (carbetocin OR pabal OR depotocin OR duratocin OR lonacetene). The actual strategy employed in each database is shown in the Appendices. We also checked the bibliographies of the papers we had gathered in order to broaden the scope of the literature study. Moreover, we excluded trials whose collected information could not be regarded for analysis. Two authors thoroughly examined the titles and abstracts of each related study found in the resources, eliminated duplication, and assessed validity by full-text screening. Additionally, the eventually selected papers' citations were personally checked for any new or missing sources. Conflicts were resolved through discussions.

Extraction of Data and Evaluation of Study Quality

To rate the quality of the articles that were included, we used the Cochrane Risk of Bias checklist (version 2) [18]. Two authors individually evaluated this work. Each scale domain and the overall quality of the chosen publications were given a risk level from low, with some concerns, to high by the authors. Conflicts were resolved through discussions. For combined research with less than 10 investigations, public bias is unreliable. As a result, we were unable to utilize Egger's test [19].

The first three types of data were gathered. In the beginning, we made a list of the features of the investigations that were included, such as the trial identification, country, duration, sample size, and research arms. Second, we obtained data on the fundamental details of the participants, such as sample size, age (years), gestational age (in weeks), parity, body mass index (BMI), delivery technique, and anesthesia type. Third, we collected data on effectiveness results, including intraoperative blood loss (ml), mean change in hemoglobin (mg/dl), and mean change in hematocrit (%). Also, we gathered information on blood transfusion need, additional uterotonics need, and additional need for surgical interventions (like uterine artery ligation or compressed suturing). Moreover, we collected information on safety profiles such as headache, fever, heat sensation, palpitations, metallic taste, and shivering.

Statistical Analysis

The Review Manager program, available for Windows as version 5.4 of RevMan (The Cochrane Collaboration, 2020), was used for data analysis. We combined the dichotomous and continuous data under the random-effects model for calculating the risk ratio (RR) and mean difference (MD) with a 95% confidence interval (Cl). We depended on the Inverse-Variance and Mantel-Haenszel techniques for our analyses. Heterogeneity was assessed by utilizing the chi-square tests. Significant heterogeneity was determined when the chi-square test with p<0.1 and the I2 test >50 [20]. A p-value of 0.05 or lower is considered statistically substantial.

Results

Results of the Literature Search

**Figure 1:**
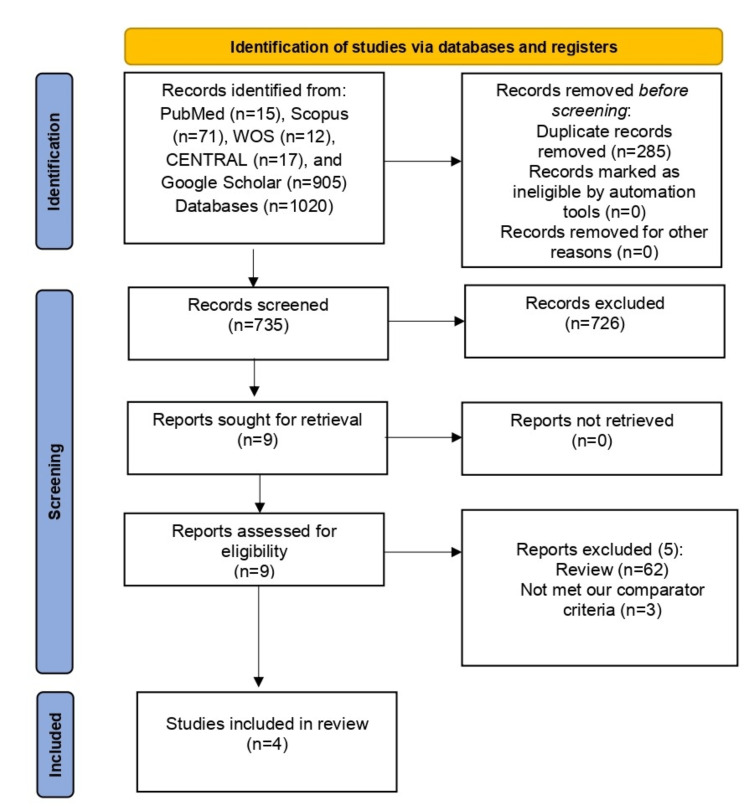
PRISMA flow diagram PRISMA: Preferred Reporting Items for Systematic Reviews and Meta-Analyses

After excluding 285 duplicate articles, our search returned 735 articles. After that, during title/abstract screening, 726 references were eliminated. Finally, following the exclusion of 10 articles during full-text screening, four RCTs [14,21-23] satisfy our Population, Intervention, Comparison, Outcomes, and Study (PICOS) requirements. The PRISMA flowchart for our screening procedure is shown in Figure 1. Seven hundred patients participated in these investigations, 305 were administered misoprostol and 305 were administered carbetocin.

Study Characteristics

Although the length and trial settings varied, all included RCTs were conducted in Egypt. Most RCTs [14,21,23] used misoprostol per rectum except one [22], which used it sublingually. Also, three RCTs [14,21,22] operated on patients under spinal anesthesia, and one RCT [23] performed on their patients under general anesthesia. Table 1 and Table 2 depict a summarization of the baseline features of the eligible articles.

**Table 1:** Summary of the included trials [14,21-23]

Study ID	Country	Trial duration (hospital)	Total sample size, n	Study arms	
				Intervention	Control
Ali 2018 [14]	Egypt	October 2016-August 2017 (Qena and Al-Azhar University Hospitals)	n=100	Carbetocin (100 mg, IV)	Misoprostol (400 μg, rectally)
Elbohoty 2016 [22]	Egypt	October 2012-June 2013 (Ain-Shams University Maternity Hospital)	n=180	Carbetocin (100 μg/mL, IV)	Misoprostol (400 μg, sublingual)
Elgazayerli 2019 [21]	Egypt	Not reported (El-Shatby Maternal Hospital)	n=120	Carbetocin (100 mg, IV)	Misoprostol (400 μg, rectally)
Moustafa 2020 [23]	Egypt	February 2017-February 2018 (Mansoura University Hospitals)	n=300	Carbetocin (100 μg/mL, IV)	Misoprostol (600 μg, sublingual)

**Table 2:** Baseline characteristics of the included trials [14,21-23]

Study ID		Group	Sample size, n	Age (years)	Gestational age (weeks)	Parity	BMI (kg/m²)	Type of delivery	Type of anesthesia	
										Ali 2018 [14]		Carbetocin	n=50	27.82 ±4.6	38.2 ±0.90	2.3 ±1.81	26.95 ±3.8	Cesarean section	Spinal	
Misoprostol	n=50	28.2 ±3.6	38.7 ±0.73	2.3 ±1.78	26.95 ±3.8														
Elbohoty 2016 [22]		Carbetocin	n=90	28.0 ±5.5	38.4 ±0.8	2.5 ±1.7	33.0 ±5.2	Cesarean section	Spinal	
		Misoprostol	n=90	27.9 ±5.2	38.4 ±0.8	3 ±2	32.8 ±5.4			
Elgazayerli 2019 [21]		Carbetocin	n=60	25.6 ±3.98	38.8 ±0.67	1.00 ±0.38	27.9 ±3.85	Cesarean section	Spinal	
		Misoprostol	n=60	24.6 ±5.21	39.1 ±0.77	1.03 ±0.41	28.1 ±4.01			
Moustafa 2020 [23]		Carbetocin	n=150	28.06 ±4.3	Not reported	2.5 ±1.7	Not reported	Cesarean section	General	
		Misoprostol	n=150	28.59 ±4.8	Not reported	2 ±1.4	Not reported			

Quality Assessment of Studies

**Figure 2:**
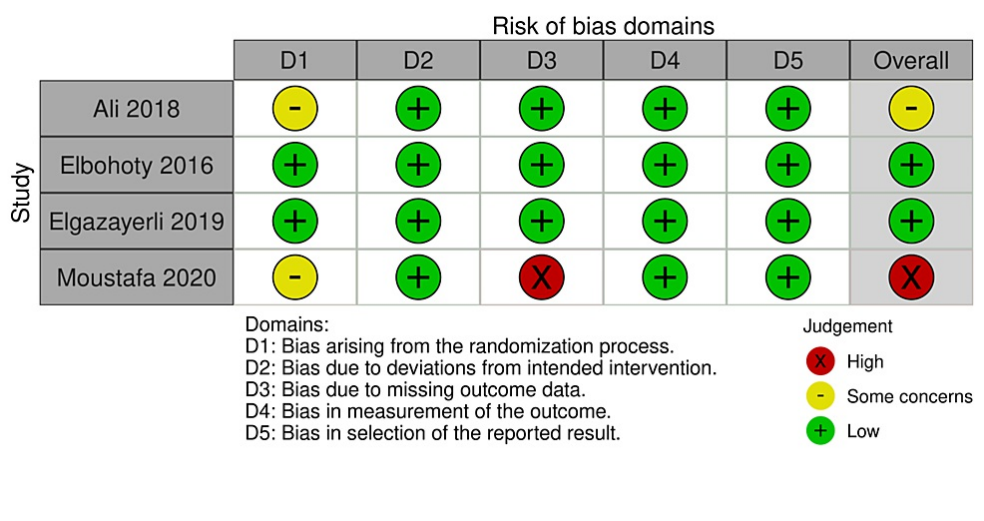
Risk of bias summary [14,21-23]

**Figure 3:**
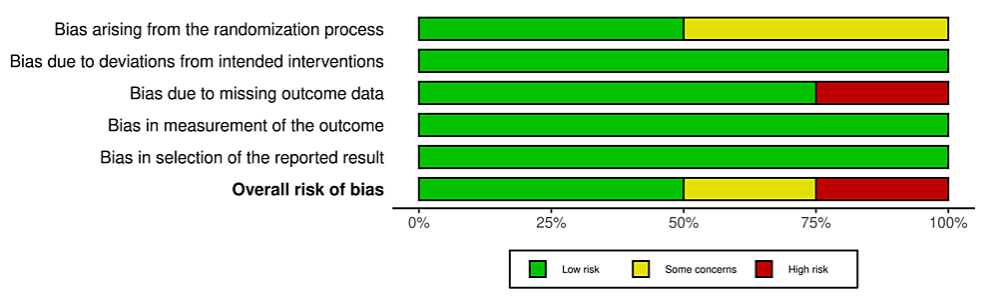
Risk of bias graph [14,21-23]

Figure 2 and Figure 3 show the quality assessment of the eligible RCTs - two RCTs [21,22] were assessed as having a “low” risk of bias. However, one RCT [14] was considered as having “some concerns” risk of discrimination because it did not provide any data about the process of randomization, one RCT [23] did not report an important outcome such as blood loss estimation, and there are no postoperative values for hemoglobin and hematocrit to assess the mean change between groups.

Results of the Meta-Analysis

**Figure 4:**
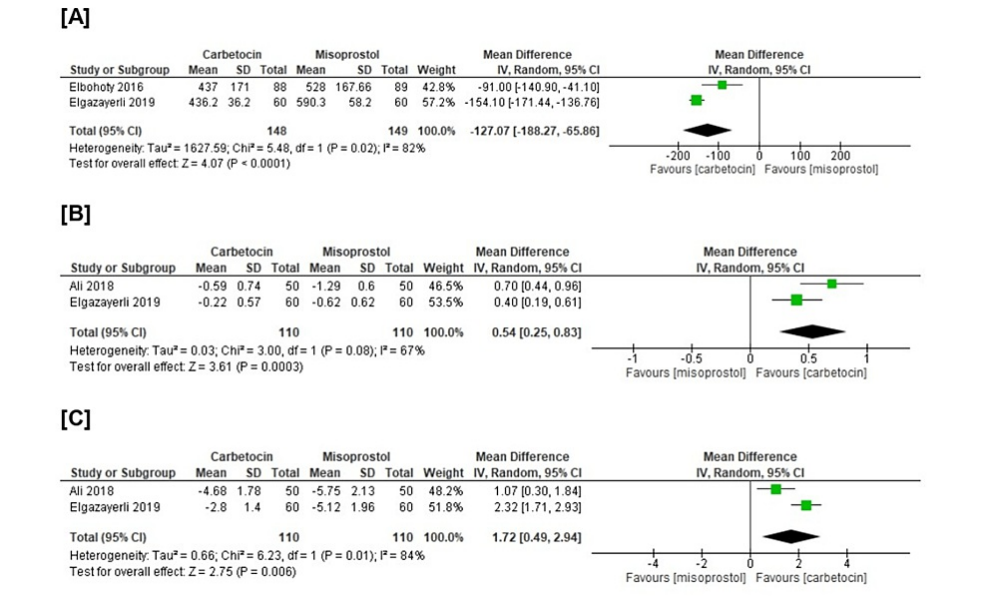
Meta-analysis of the (A) intraoperative blood loss (ml), (B) mean change in hemoglobin (mg/dl), and (C) mean change in hematocrit (%) [14,21-22]

From the efficacy prospects, we revealed a substantial difference that favors carbetocin over misoprostol concerning intraoperative blood loss (n=2 RCTs, MD=-127.07, CI 95% (-188.27, -65.86), p<0.001), mean change in hemoglobin (n=2 RCTs, MD=0.54, 95% CI [0.25, 0.83], p<0.001), and in mean change in hematocrit (n=2 RCTs, MD=1.72, 95% CI [0.49, 2.94], p<0.001). All gathered analyses were heterogeneous (chi-square p<0.1, I-square>50%) (Figure 4).

**Figure 5:**
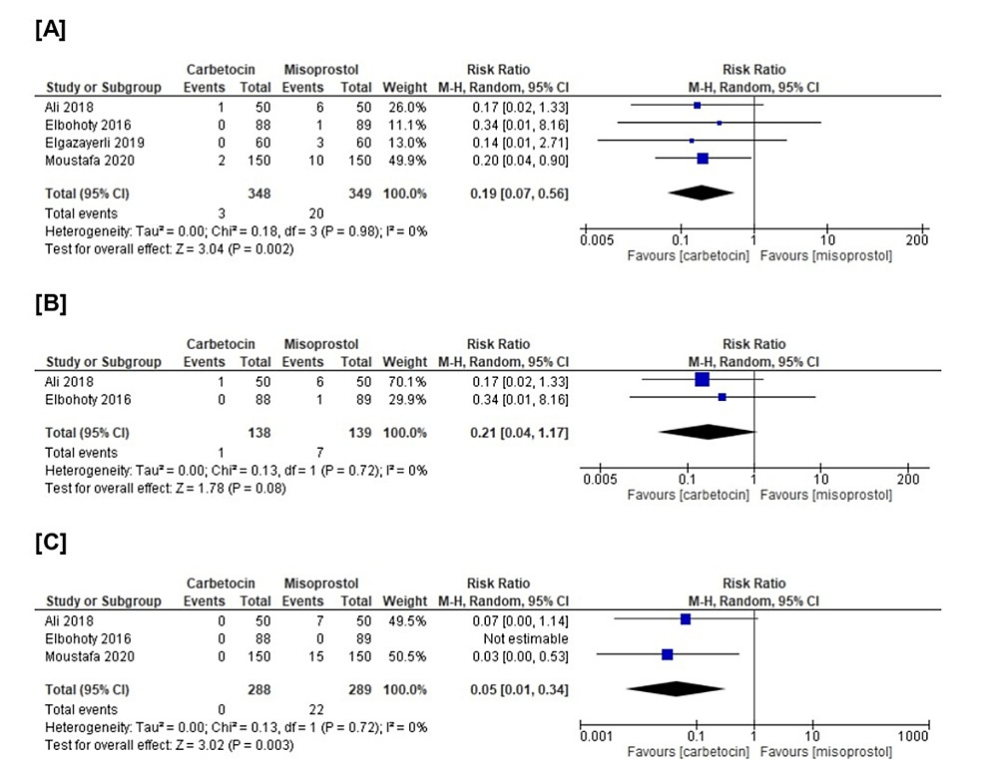
Meta-analysis of the (A) need for blood transfusion (%), (B) need for additional uterotonic agents, and (C) need for additional surgical interventions [14,21-23]

Also, we revealed a substantial difference that favors carbetocin over misoprostol in those in need of blood transfusion (n=4 RCTs, RR=0.19, 95% CI [0.07, 0.56], p=0.002) and in need of additional surgical interventions (n=3 RCTs, RR=0.05, 95% CI [0.01, 0.34], p=0.003). On the other hand, we revealed no substantial difference between both groups in need of uterotonic agents (n=2 RCTs, RR=0.21, 95% CI [0.04, 1.17], p=0.08). All the pooled analyses were homogenous (chi-square p>0.1, I-square<50%) (Figure 5).

**Figure 6:**
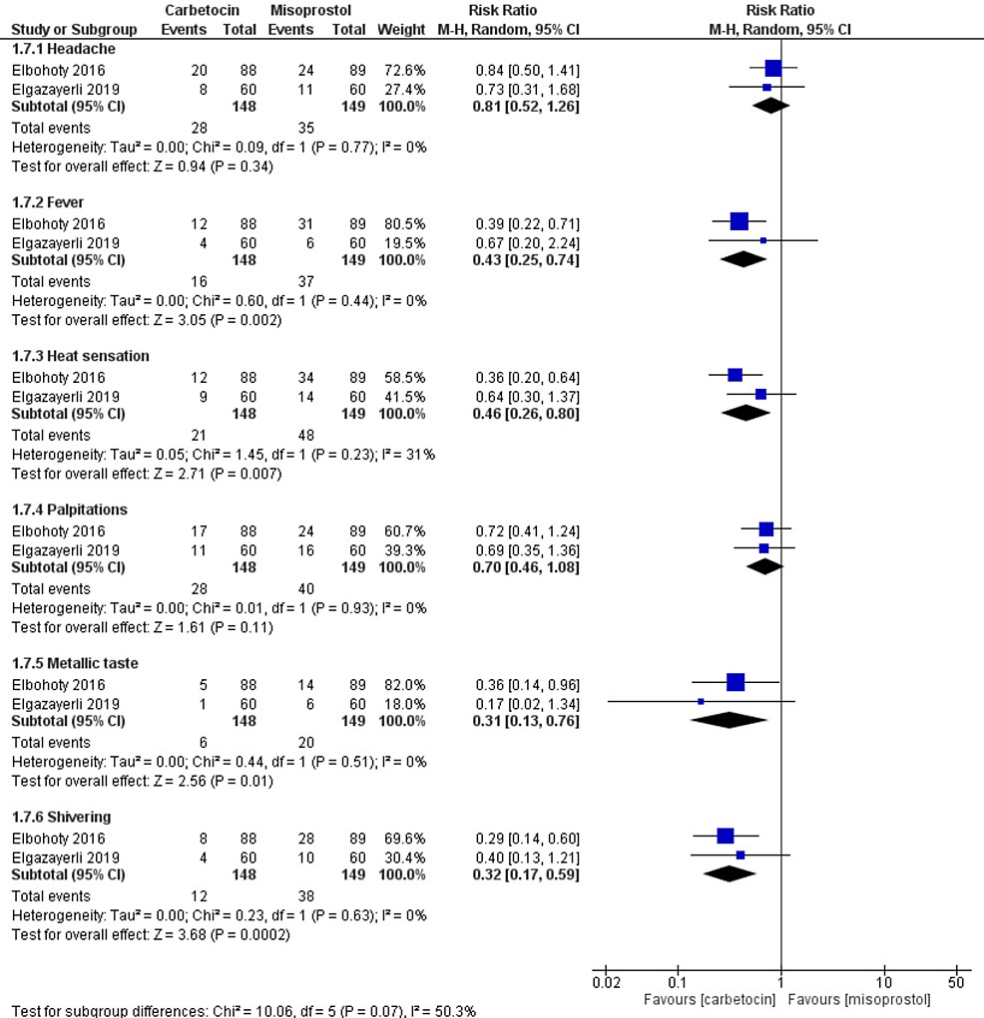
Meta-analysis of the safety endpoints (headache, fever, heat sensation, palpitations, metallic taste, and shivering) [21,22]

Regarding safety endpoints, there was a significant difference that favors the carbetocin group compared with the misoprostol group in the incidence of fever (n=2 RCTs, RR=0.43, CI 95% [0.25, 0.74], p=0.002), the incidence of heat sensation (n=2 RCTs, RR=0.46, CI 95% [0.26, 0.80], p=0.007), the incidence of metallic taste (n=2 RCTs, RR=0.31, CI 95% [0.13, 0.76], p=0.01), and the incidence of shivering (n=2 RCTs, RR=0.32, CI 95% [0.17, 0.59], p=0.0002). However, there were insignificant variations between the carbetocin group and the misoprostol group in the incidence of headache (n=2 RCTs, RR=0.81, CI 95% [0.52, 1.26], p=0.34), and the incidence of palpitations (n=2 RCTs, RR=0.70, CI 95% [0.46, 1.08], p=0.11). All the gathered analyses were homogenous (chi-square p>0.1, I-square<50%) (Figure 6).

Discussion

Increased CS practice, particularly for non-medical reasons, carries numerous short-term hazards to the mother, including PPH, blood transfusion, hysterectomy, and maternal mortality [24,25]. In an era of rising CS rates, particularly in Egypt, numerous measures should be taken to reduce maternal comorbidities, such as lowering PPH and requiring blood transfusions.

Our study evaluated carbetocin's effectiveness and adverse events versus misoprostol in preventing PPH in women undergoing CS. From the efficacy prospect, we found that carbetocin substantially decreased intraoperative blood loss, hemoglobin/hematocrit levels, and the need for blood transfusion/additional surgical interventions than misoprostol. However, we revealed no substantial variation between both drugs for the need for additional uterotonic agents. From the safety prospect, we found that incidences of fever, heat sensation, metallic taste, and shivering were lower in the carbetocin administration than in misoprostol. However, headache and palpitation incidences revealed no substantial variation between both drugs.

Our results were consistent with a prior meta-analysis that contrasted rectal misoprostol with carbetocin for vaginal birth. They discovered that carbetocin was linked to less blood loss and a decreased requirement for blood transfusions [26]. These results are consistent with those of Abd El Aziz et al. and Hetiba et al., who discovered that women who birthed vaginally or via CS experienced much less blood loss in the carbetocin administration versus the misoprostol [27,28]. Even during other surgeries, such as myomectomies, the introduction of carbetocin was associated with many favorable clinical effects, such as a reduction in operation bleeding and the requirement for blood transfusions [29].

Furthermore, in a recent RCT, authors compared the administration of Carbetocin versus syntocinon and misoprostol for women during CS. They discovered that hemoglobin and hematocrit levels 24 hours postoperatively showed a moderately substantial change among the three examined groups [21]. Therefore, carbetocin protects or reduces the incidence of post-CS hemorrhagic anemia.

A thorough analysis revealed that the misoprostol group experienced severe placental bleeding more frequently than the carbetocin group did, as shown by the existence of a floppy uterus after the birth of the fetus and placenta [23]. This is mainly related to carbetocin's strong uterotonic impact, which was demonstrated in a prior study by Cordovani and colleagues, who discovered that it lowers the rate of uterine atony in low-risk women [30].

Additional surgical procedures, such as uterine artery ligation and uterine compression stitching, are required to reduce bleeding from uterine atony, which was more severe in the misoprostol arm [23]. This is consistent with our research, which showed that carbetocin minimizes the need for further surgical procedures to reduce bleeding during CS.

The medication type used in the current investigation as prevention against PPH had no influence on the need for uterotonic drugs, as we revealed no substantial variation between both drugs for the need for additional uterotonic agents. This was in contrast with Su et al., who discovered that carbetocin considerably reduces the requirement for additional uterotonic medications [15]. Ali et al. also found that uterine atony, which was more pronounced in the misoprostol group, necessitated the usage of more oxytocin [14].

The adverse effects in our investigation led to a range of results; some were statistically meaningful while others had no influence. We found that incidences of fever, heat sensation, metallic taste, and shivering were lower in carbetocin administration than in misoprostol. However, headache and palpitation incidences revealed no substantial variation between both drugs. Another research discovered a substantial variation between the misoprostol and carbetocin arms in terms of the number of patients who suffered from side effects like fever, nausea, diarrhea, and stomach pain after delivery; the participants in the carbetocin arm were less likely to suffer such adverse reactions. However, when there were negative effects such as hypersensitivity, face flushing, and headaches, it was shown that there was no scientifically substantial variation between the arms taking carbetocin and misoprostol [28].

The findings of several investigations on the adverse effects of the drugs utilized to prevent PPH reported significant inconsistencies and are inconsistent with one another due to multiple variables connected to demographic and population variables. For instance, Abd El-Aziz et al. found that misoprostol had worse side effects than carbetocin in aspects of heart rate and feeling hot [27]. Additionally, Ibrahim and Saad reported the results of their investigation into side effects, concluding that carbetocin was more usually associated with headache, nausea, and vomiting. Misoprostol was also more frequently associated with pyrexia and shivering [31].

Limitations

There are some concerns with the current study. The main weak point was that there weren't many trials that were included, which prevented us from investigating publication bias. Also, not all outcomes were homogeneous, as we found heterogeneity in some results. The participants' brief follow-up intervals and certain researchers' or subjects' lack of blinding were further drawbacks.

## Conclusions

From the efficacy and safety perspective, for women who underwent CS, carbetocin is more effective and safer in preventing and reducing PPH than misoprostol in terms of decreased intraoperative blood loss, hemoglobin/hematocrit levels, and the need for blood transfusion/additional surgical interventions than misoprostol. However, we revealed no substantial variation between both drugs for the need for additional uterotonic agents. From the safety prospect, we found that incidences of fever, heat sensation, metallic taste, and shivering were lower in carbetocin administration than in misoprostol. However, headache and palpitation incidences revealed no substantial variation between both drugs. It is crucial to proceed cautiously while considering this conclusion because the assessment of PPH is often a subjective judgment. Future trials that employ other administration techniques, evaluation of several doses, and assessment of the impact of those drugs are required to verify our investigations.

## Appendices

**Table 3:** The exact literature search strategy used in every database

PubMed All Fields: (“cesarean section” OR CS OR “C-section” OR “abdominal delivery”) AND (misoprostol OR “novo misoprostol” OR “apo misoprostol” OR cytotec OR “SC30249” OR “SC29333” OR glefos OR misodel OR mysodelle OR misotac) AND (carbetocin OR pabal OR depotocin OR duratocin OR lonacetene).
Scopus Article title, Abstract, Keywords: (“cesarean section” OR CS OR “C-section” OR “abdominal delivery”) AND (misoprostol OR “novo misoprostol” OR “apo misoprostol” OR cytotec OR “SC30249” OR “SC29333” OR glefos OR misodel OR mysodelle OR misotac) AND (carbetocin OR pabal OR depotocin OR duratocin OR lonacetene).
Web of Science All Fields: (“cesarean section” OR CS OR “C-section” OR “abdominal delivery”) AND (misoprostol OR “novo misoprostol” OR “apo misoprostol” OR cytotec OR “SC30249” OR “SC29333” OR glefos OR misodel OR mysodelle OR misotac) AND (carbetocin OR pabal OR depotocin OR duratocin OR lonacetene).
Cochrane CENTRAL Title Abstract Keyword: (“cesarean section” OR CS OR “C-section” OR “abdominal delivery”) AND (misoprostol OR “novo misoprostol” OR “apo misoprostol” OR cytotec OR “SC30249” OR “SC29333” OR glefos OR misodel OR mysodelle OR misotac) AND (carbetocin OR pabal OR depotocin OR duratocin OR lonacetene).
Google Scholar All Fields: (“cesarean section” OR CS OR “C-section” OR “abdominal delivery”) AND (misoprostol OR “novo misoprostol” OR “apo misoprostol” OR cytotec OR “SC30249” OR “SC29333” OR glefos OR misodel OR mysodelle OR misotac) AND (carbetocin OR pabal OR depotocin OR duratocin OR lonacetene).

## References

[1] Villar J, Valladares E, Wojdyla D (2006). Caesarean delivery rates and pregnancy outcomes: the 2005 WHO global survey on maternal and perinatal health in Latin America. Lancet.

[2] Betran AP, Torloni MR, Zhang JJ, Gulmezoglu AM (2016). World Health Organization statement on caesarean section rates. BJOG.

[3] El-Zanati F (2015). El-Zanati F. Egypt health issues survey 2015. Ministry of Health and Population, Cairo, Egypt. by: Economic and Social Justice Unit: Knowledge and Prevalence of Hepatitis B and C.

[4] (2012). WHO recommendations for the prevention and treatment of postpartum haemorrhage. Evidence base. https://apps.who.int/iris/bitstream/handle/10665/75519/WHO_RHR_12.29_eng.pdf.

[5] McLintock C (2020). Prevention and treatment of postpartum hemorrhage: focus on hematological aspects of management. Hematology Am Soc Hematol Educ Program.

[6] Ismail IAEM, Fahmy MSED, Farouk HA, Ismail IAEM (2019). Carbetocin versus misoprostol in prevention of postpartum hemorrhage in high risk patients. Egypt J Hosp Med.

[7] Oladapo OT, Fawole B, Blum J, Abalos E (2012). Advance misoprostol distribution for preventing and treating postpartum haemorrhage. Cochrane Database Syst Rev.

[8] de Castro Parreira MV, Gomes NC (2013). Preventing postpartum haemorrhage: active management of the third stage of labour. J Clin Nurs.

[9] Mobeen N, Durocher J, Zuberi N (2011). Administration of misoprostol by trained traditional birth attendants to prevent postpartum haemorrhage in homebirths in Pakistan: a randomised placebo-controlled trial. BJOG.

[10] Gülmezoglu AM, Forna F, Villar J, Hofmeyr GJ (2007). Prostaglandins for preventing postpartum haemorrhage. Cochrane Database Syst Rev.

[11] Tang OS, Gemzell-Danielsson K, Ho PC (2007). Misoprostol: pharmacokinetic profiles, effects on the uterus and side-effects. Int J Gynaecol Obstet.

[12] Awoleke JO, Adeyanju BT, Adeniyi A, Aduloju OP, Olofinbiyi BA (2020). Randomised controlled trial of sublingual and rectal misoprostol in the prevention of primary postpartum haemorrhage in a resource-limited community. J Obstet Gynaecol India.

[13] Lucas DN, Meshykhi LS, Nel MR (2017). The role of carbetocin in the prevention and management of postpartum haemorrhage - in reply. Int J Obstet Anesth.

[14] Abd El-Gaber AE-N, Ali AAM, Ahmed HH, El-Rasheedy MI, Badawy M (2018). Carbetocin versus oxytocin and misoprostol in prevention of atonic post-partum hemorrhage in high risk patients planed for cesarean delivery. Int J Reprod Contracept Obstet Gynecol.

[15] Su LL, Chong YS, Samuel M (2012). Carbetocin for preventing postpartum haemorrhage. Cochrane Database Syst Rev.

[16] Page MJ, McKenzie JE, Bossuyt PM (2021). The PRISMA 2020 statement: an updated guideline for reporting systematic reviews. Syst Rev.

[17] Higgins JPT, Green S (Sally E, Collaboration Collaboration (2008). Guide to the contents of a Cochrane protocol and review. Cochrane Handbook for Systematic Reviews of Interventions: Cochrane Book Series.

[18] Sterne JA, Savović J, Page MJ (2019). RoB 2: a revised tool for assessing risk of bias in randomised trials. BMJ.

[19] Egger M, Davey Smith G, Schneider M, Minder C (1997). Bias in meta-analysis detected by a simple, graphical test. BMJ.

[20] Higgins JP, Thompson SG, Deeks JJ, Altman DG (2003). Measuring inconsistency in meta-analyses. BMJ.

[21] Elgazayerli WS (2019). Comparison between syntocinon, misoprostol and carbetocin in reducing blood loss in elective caesarean section. Journal of Evidence Based Women’s Health.

[22] Elbohoty AE, Mohammed WE, Sweed M, Bahaa Eldin AM, Nabhan A, Abd-El-Maeboud KH (2016). Randomized controlled trial comparing carbetocin, misoprostol, and oxytocin for the prevention of postpartum hemorrhage following an elective cesarean delivery. Int J Gynaecol Obstet.

[23] Moustafa A, Abd Elhady S, Shalaby H, Elrefaie W (2020). Carbetocin versus Misoprostol in Reducing Blood Loss during Cesarean Section in low risk patients. A Randomized Controlled Trial. Journal of Evidence Based Women’s Health.

[24] Rossen J, Økland I, Nilsen OB, Eggebø TM (2010). Is there an increase of postpartum hemorrhage, and is severe hemorrhage associated with more frequent use of obstetric interventions?. Acta Obstet Gynecol Scand.

[25] Souza JP, Gülmezoglu A, Lumbiganon P, Laopaiboon M, Carroli G, Fawole B, Ruyan P (2010). Caesarean section without medical indications is associated with an increased risk of adverse short-term maternal outcomes: the 2004-2008 WHO Global Survey on Maternal and Perinatal Health. BMC Med.

[26] Albazee E, Alrashidi H, Laqwer R (2022). Intravenous carbetocin versus rectal misoprostol for the active management of the third stage of labor: a systematic review and meta-analysis of randomized controlled trials. Cureus.

[27] Abd El Aziz MA, Iraqi A, Abedi P, Jahanfar S (2018). The effect of carbetocin compared to misoprostol in management of the third stage of labor and prevention of postpartum hemorrhage: a systematic review. Syst Rev.

[28] Abdo Mohamed Hetiba YA-E, Mahmoud M, Oun AEM (2021). Carbetocin versus rectal misoprostol to decrease blood loss in vaginal delivery in high risk patients for postpartum hemorrhage. Int J Med Arts.

[29] Albazee E, Sayad R, Elrashedy AA, Samy Z, Faraag E, Baradwan S, Samy A (2022). Efficacy of oxytocics on reducing intraoperative blood loss during abdominal myomectomy: a systematic review and meta-analysis of randomized placebo-controlled trials. J Gynecol Obstet Hum Reprod.

[30] Cordovani D, Balki M, Farine D, Seaward G, Carvalho JC (2012). Carbetocin at elective cesarean delivery: a randomized controlled trial to determine the effective dose. Can J Anaesth.

[31] Ibrahim KAAM, Saad AS (2017). Prevention of postpartum haemorrhage in patients with severe preeclampsia using carbetocin versus misoprostol. Apollo Medicine.

